# Immediate newborn care of knowledge, practice and associated factors among health care providers in Northwestern Zonal health facilities Tigray, Ethiopia, 2018

**DOI:** 10.1186/s13104-019-4465-z

**Published:** 2019-07-17

**Authors:** Hagos Tasew, Tsega Teshale, Degena Bahrey, Teklewoini Mariye, Girmay Teklay

**Affiliations:** 1 College of Health Science and Comprehensive Specialized Hospital, School of Nursing, Aksum University, Tigray, Po Box: 298, Aksum City, Ethiopia; 2 College of Health Science and Comprehensive Specialized Hospital, Department of Medical Laboratory, Aksum University, Tigray, Po Box: 298, Aksum City, Ethiopia

**Keywords:** Knowledge, Practice, Immediate newborn care, Northwest, Ethiopia

## Abstract

**Objective:**

The aim of this study was to assess the immediate newborn care of knowledge, practice and associated factors among healthcare providers in Northwestern Zonal health facilities Tigray, Ethiopia, 2018.

**Results:**

Among the total healthcare providers, who participated in this study, 64.8% had good knowledge and 59.8% of the respondents had a good level of essential newborn care practice. Unavailability of adequate materials (like guidelines, drug, etc.) and training status were significant variables with knowledge and practice of newborn care.

## Introduction

The first 28 days of life in a newborn is the most susceptible time for a child’s survival [1]. Globally, 2.5 million children died in the first month of life. One million died on the first day of life [2]. The rate of neonatal, infant and under-5 mortality were 29, 48, and 67 deaths per 1000 live births, respectively in Ethiopia. Which means that one in every 35 children dies within the first month, one in every 21 children dies before the first year, and one of every 15 children dies before reaching the fifth year [3]. Poor mother’s healthcare behavior and high rate of home-based delivery are the most likely reasons for many deaths of newborns in Ethiopia [4].

Even though 70% of infant deaths happen during the first month of life, most sub-Saharan countries neglected the care of the newborn because of little attention to a high number of deaths [5].

World Health Organization (WHO) was planned to reduce neonatal mortality to below 12 per 1000 live births by 2030 [6]. However, healthcare providers have poor knowledge and practice regarding prenatal and newborn care [7].

General nurses, midwives, and nursing assistants are routinely positioned to care newborns admitted at the formal health facilities [8]. Therefore, the enhancement of their knowledge and skills are very vital aspects of these health facilities [9]. Morbidity and mortality of newborns are reduced by improving access to educational messages and treatments of women [10]. Therefore, appropriate care of newborns is crucial for the survival, growth, and development of newborns [11].

Consequently, Ethiopia has high perinatal mortality and morbidity. There is little known on the knowledge, practice and associated factors of immediate newborn care among healthcare providers in Ethiopia. Therefore, the aim of this study was to assess the knowledge, practice and associated factors of immediate newborn care among healthcare providers in Northwestern Zonal health facilities Tigray, Ethiopia, 2018.

## Main text

The facility-based cross-sectional study design was used in governmental public health facilities of the Northwestern Zone of Tigray. The study period was from January to March 2018. The source population was all nurses who were working in the study area and the study population was all selected nurses from the selected health facilities.

### Sample size and sampling technique

Single population proportion formula was used and the following assumptions were made: 73% of good essential immediate newborn care practice from the previous studies conducted in the Eastern Zone of Tigray [12], 5% marginal error (d) with 95% confidence was employed as a parameter. Because the source population is less than 10,000, sample size correction was made. By assuming correction formula and 10%, non-response rate the final sample size was 179 nurses and midwives. Simple random sampling technique was used to select the study participants. The sample size was proportionally allocated to each public health facilities.

### Data collection tools and procedures

An interviewer-administered structured and pre-tested questionnaire was used to collect the knowledge of immediate newborn care and an observational checklist for the practice of immediate newborn care. The questionnaire was adopted from other published articles [12–14] and further modification was done to fit the local context and research objective based on the Ethiopian Federal Ministry of health newborn care training participants manual [15]. Three BSc holder nurses were recruited for data collection and two MSc holder midwives were recruited as a supervisor. Overall, the data collection process was coordinated and supervised by the principal investigator.

### Study variables

The dependent variable was the knowledge and practice of health care providers on immediate newborn care. The independent variables were socio-demographic factors and personal and institutional factors.

### Operational definitions

#### Good knowledge

If the health care provider answers the knowledge question above or equal to mean [12].

#### Poor knowledge

If the health care provider answers the knowledge questions below mean [12].

#### Good practice

If the health care providers perform more than or equal to 70% the practice procedures [12].

#### Poor practice

If the health care providers perform less than 70% of the practice procedures [12].

### Data quality control

Data collectors trained about the aim of study and methods of data collection. English version of the questionnaire was prepared and translated to Tigrigna version (local language). The tool was pre-tested on five percent of the sample size in St. merry hospital of Aksum. Supervisor and principal investigator made continuous follow-up and supervision for completeness and consistency of the data.

### Data processing and analysis

Epi Data manager was used for cleaning and entering of data and then exported to SPSS Version 22.0 for analysis. The knowledge questions were computed and graded as 1 and 0 and dichotomized as good knowledge and poor knowledge. The practice of essential newborn care was graded by assigning scores to Likert scale responses on a scale of 1–3 points: 1 = never, 2 = sometimes, 3 = always and dichotomized into 1 and 0 based on the summed score considering 70% score as the cut-off point.

A binary logistic regression model was used to test the statistical association between the outcome variable and independent variables. Significance variables at p-value ≤ 0.2 were entered into binary logistic regression. The goodness-of-fit of the model was checked by the Hosmer–Lemeshow test. Finally, statistical significance was declared at a p-value < 0.05.

## Results

### Socio-demographic and institutional characteristics of health care providers of the Northwest Zone of Tigray

In this study, 179 participants consented to participate in the study with a 100% response rate. The largest proportion, 142 (79.3%) of the respondents were between the ages of 20 and 35 years. Mostly, 95 (53.1%) of the respondents were single. One hundred sixty-nine (91.6%) were orthodox and 96 (53.6%) were a degree holder, 94 (52.5%) were midwifery profession. Almost half of the participants worked in health center 90 (50.3%). One hundred nineteen (66.5%) of participants were trained about newborn care within the past two years. About 121 (67.6%) of health professionals had equipment’s for immediate newborn care. One hundred twenty-six (70.4%) of the study participants had enough drugs and vaccines for caring the newborns (Table 1).

**Table 1:** Socio-demographic and institutional characteristics of health care providers in northwestern zone health facilities Tigray, Ethiopia, 2018

Variable	Frequency	Percentage
Age		
1. 20–35	142	79.3
2. 36–46	29	16.2
3. > 46	8	4.5
Sex		
1. Male	80	44.7
2. Female	99	55.3
Religion		
1. Orthodox	164	91.6
2. Muslim	15	8.4
Educational status		
1. Degree	96	53.6
2. Diploma	83	46.4
Marital status		
1. Married	80	44.7
2. Single	95	53.1
3. Divorced	4	2.2
Field of study		
1. Nurse	85	47.5
2. Midwifery	94	52.5
Monthly salary		
1. 1946–2406	10	5.6
2. 2500–3114	54	30.2
3. 3214–4446	73	40.8
4. > 4446	42	23.5
Type health facility		
1. Hospital	89	49.7
2. Health center	90	50.3
Working experience (in the year)		
1. 0–2 years	76	42.5
2. 2–4 years	57	31.8
3. > 4 years	46	25.7
Workload		
1. Yes	155	86.6
2. No	24	13.4
Training on immediate newborn care		
1. Yes	119	66.5
2. No	60	33.5
How many times		
1. One	65	54.6
2. Two	31	26.1
3. Three	23	19.3
Availability of equipment		
1. Yes	121	67.6
2. No	58	32.4
Availability of drugs and vaccines		
1. Yes	126	70.4
2. No	53	29.6

### Knowledge and practice of health care providers on essential newborn care

The overall mean knowledge score of the health care providers was 40.91 (SD =  ± 3.88). In this study, 116 (64.8%) of the respondents had good knowledge. One hundred twenty (67%) of participants started newborn care immediately after birth. Ninety-three (52%) of the participants used skin-to-skin contact to prevent hypothermia. About 137 (76.5%) of the respondents' used a bag and mask for ventilation. Concerning the breastfeeding, about 137 (76.5%) of the participants reported that breastfeeding should be initiated within the first hour of birth. One hundred fifty-six (87.2%) of the respondents agreed that colostrum has infection prevention role for the child. Sixty-five (36.3%) of study participants agreed on the umbilical cord should tie immediately after birth. One hundred twenty-two (68.2%) used sterile Scissor to cut the umbilical cord.

The mean score of the practice of ENC was 41.35 (SD =  ± 4.65). One hundred seven (59.8%) of the respondents had a good level of practice. During observation of newborn care practice, 118 (65.9%) of our study participants washed their hand before the procedure. One hundred thirty-four (74.9%) used a sterile glove, 132 (73.7%) used an apron, and 108 (60.3%) used a mask during the procedure of newborn care practice.

One hundred twenty-six (70.4%) of the study participants wiped eye & face immediately after delivery. One hundred fifty (83.8%) dried the baby immediately with a dry towel. About 151 (84.4%) study participants performed skin-to-skin contact with the caregiver of the newborn and 158 (88.3%) were weighed and recorded the baby’s weight. About 157 (87.7%) of the participants were counseled mother about danger sign of newborns before discharge (Table 2).

**Table 2:** Knowledge and practice of health care providers on essential newborn care in northwestern zone health facilities Tigray, Ethiopia, 2018

Variable	Frequency	Percentage
*Knowledge variables*		
When starting ENBC		
1. Before birth	15	8.4
2. During birth	42	23.5
3. After birth	120	67.0
4. I don’t know	2	1.1
After birth newborn kept on		
1. Beside the mother	50	27.9
2. With someone else	7	3.9
3. On the mother’s chest/ belly	108	60.3
4. On newborn bed /table	14	7.8
Method to prevent hypothermia		
1. Immediately drying	61	34.1
2. Allowing skin to skin contact	93	52.0
3. Early bathing	16	8.9
4. Other	9	5.0
Measures if the baby not cries		
1. Cover the baby and allow the skin to skin contact	35	19.6
2. Call a help and start resuscitation	123	68.7
3. Put bay on the newborn table and give mother care	16	8.9
4. Other	5	2.8
The position of the baby’s head to help open the airway		
1. A flexed position of the head	33	18.4
2. The slightly extended position of head	135	75.4
3. Hyperextend extended position of head	9	5.0
4. Other	2	1.1
The mentioned measures if the baby is not breathing well after stimulation		
1. More stimulation to breath	39	21.8
2. Ventilation with bag and mask	137	76.5
3. Other	3	1.7
Recommended breath per minutes during ventilation		
1. 30 breaths per minute	34	19.0
2. 40 breaths per minutes	86	48.0
3. 60 breaths per minute	59	33.0
Initiation of breastfeeding after birth		
1. After 6 h of delivery	18	10.1
2. Within 1–6 h of delivery	23	12.8
3. Within the first hour of delivery	137	76.5
4. Other	1	.6
How long EBF		
1. Less than 6 month	14	7.8
2. For 6 months	130	72.6
3. Greater 6 months	35	19.6
Colostrum has infection protection role		
1. Yes	156	87.2
2. No	23	12.8
Time to clamp or tie the umbilical cord of a crying baby		
1. Clamp or tie immediately	65	36.3
2. Clamp or tie 1–2 min of delivery	51	28.5
3. Clamp or tie 2–3 min of delivery/after pulsation of umbilical artery stopped	63	35.2
Instrument to cut the cord		
1. Clean scissor	31	17.3
2. New surgical blade	15	8.4
3. New razor blade	9	5.0
4. Sterile scissor	122	68.2
5. Other	2	1.1
Treatment of eye infection in newborn		
1. Clean it with soap and water and cover with a bandage	13	7.3
2. Clean it with soap and water, dry it and do not cover	17	9.5
3. Use alcohol to clean the umbilicus	6	3.4
4. Other	143	79.9
Action to prevent bleeding in newborn		
1. Breastfeed the child	6	3.4
2. Not necessary to give anything	4	2.2
3. Give vitamin K	166	92.7
4. Other	3	1.7
The dose of Vitamin k for preterm babies		
1. 1 mg	35	19.6
2. 0.5 mg	139	77.7
3. Other	5	2.8
Low birth weight		
1. < 3000 gm	6	3.4
2. < 2500 gm	88	49.2
3. < 1500 gm	46	25.7
4. < 1000 gm	39	21.8
Care for low birth weight		
1. Bath often	19	10.6
2. Breastfeeding early and frequently	68	38.0
3. Keep the child warm	81	45.3
4. Prevent infection from developing	11	6.1
Time of postnatal care		
1. Within the first 24 h of delivery	117	65.4
2. On the 3rd day of delivery	29	16.2
3. On the 7th day of delivery	27	15.1
4. Other	6	3.4
*Practice variables*		
Hand washing before procedure		
1. No, never	61	34.1
2. Yes, performed	118	65.9
Put on sterile glove		
1. No, never	45	25.1
2. Yes, performed	134	74.9
Wearing apron		
1. No, never	47	26.3
2. Yes, performed	132	73.7
Wearing mask		
1. No, never	71	39.7
2. Yes, performed	108	60.3
Wipe the eye &face when the head is delivered		
1. No, never	53	29.6
2. Yes, performed	126	70.4
Dry the baby immediately with dry towel		
1. No, never	29	16.2
2. Yes, performed	150	83.8
Check & sucks the airway after delivery		
1. No, never	31	17.3
2. Yes, performed	148	82.7
Take APGAR score		
1. No, never	38	21.2
2. Yes, performed	141	78.8
Keep the baby on mother bell/ chest immediately after delivery		
1. No, never	22	12.3
2. Yes, performed	157	87.7
Skin to skin contact		
1. No, never	28	15.6
2. Yes, performed	151	84.4
Initiate breastfeeding within the first hour of delivery		
1. No, never	29	16.2
2. Yes, performed	150	83.8
Administer Vit K		
1. No, never	37	20.7
2. Yes, performed	142	79.3
Give eye ointment		
1. No, never	32	17.9
2. Yes, performed	147	82.1
Counsel mother about new bore danger before discharge		
1. No, never	22	12.3
2. Yes, performed	157	87.7
Weigh & record the baby’s weight		
1. No, never	21	11.7
2. Yes, performed	158	88.3

### Factors associated with knowledge and practice of the health professionals on immediate newborn care

In the binary logistic regression educational status, training status and availability of materials (like guidelines, drug and vaccine, etc.) were found to have a significant association with participants’ level of knowledge of ENC at a p-value of ≤ 0.2. Nevertheless, in multivariable logistic regression, unavailability of adequate materials (like guidelines, drug, etc.) was significant.

Unavailability of adequate materials 2.4 times higher to had poor knowledge (AOR = 2.4, 95% CI (1.12, 5.16)).

Similarly, workload, training status, and availability of materials (like guidelines, drug, etc.) were found to have a significant association with participants’ level of practice of ENC. However, after controlling the confounding variables only training status was significant.

Those who trained ENC 2.09 times higher to had a good level of practice (AOR = 2.09, 95% CI (1.08, 4.02)) (Table 3).

**Table 3:** Factors affecting the knowledge of the health professionals on immediate newborn care in northwestern zone health facilities Tigray, Ethiopia, 2018

Variables	Knowledge of ENBC		Crude OR (CI 95%)	Adjusted OR (CI 95%)
	Good	Poor		
Educational status				
Degree	67 (69.8%)	29 (30.2%)	1	1
Diploma	49 (59%)	34 (41%)	0.62 (0.34, 1.16)	1.5 (0.79, 2.83)
Training taking				
Yes	70 (58.8%)	49 (42.1%)	1	1
No	46 (76.7)	14 (23.3%)	2.3 (1.14, 4.6)	2.03 (0.99, 4.15)
Availability of materials (guidelines, drug, and vaccine)				
Yes	74 (58.7%)	52 (41.3%)	1	1
No	42 (79.2%)	11 (20.8%)	2.7 (1.26, 5,69)	2.4 (1.12, 5.16)*

Variables	Practice of ENBC			
	Good	Poor		
Factors associating with practice				
Workload				
Yes	96 (61.9%)	59 (38.1%)	1.9 (0.80, 4.57)	1.73 (0.70, 4.25)
No	11 (45.8%)	13 (54.2%)		
Training				
Yes	77 (64.7%)	42 (35.3%)	1.83 (0.98, 3.44)	2.09 (1.08, 4.02)*
No	30 (50%)	30 (50)	1	1
Availability of materials (guidelines, drug, and vaccine)				
Yes	71 (56.3%)	55 (43.7%)	1	1
No	36 (67.9%)	17 (32.1%)	1.64 (0.83, 3.22)	1.93 (0.95, 3.9)

*Significance association between varibles

## Discussion

The objective of this study was to assess knowledge, the practice of newborn care and associated factors among health care providers in public health facilities of the northwestern zone, Tigray, Ethiopia.

The overall knowledge of health care providers on immediate newborn care in northwestern zone of Tigray was 64.8% (57.3, 71.5). This is consistent with the study done in Addis Ababa 68% [16].

The finding of this study is higher than the studies done in Jimma 52.2% [9], in Addis Ababa public health center 51.4% [17], in Bahir Dar city 56% [18], in Khartoum 50.6% [14], in Egypt 47.9% [19], in Uganda 46.5% [7]. This difference might be due to the difference in socio-demographic and the time of the study.

However, the finding of this study is lower than the study done in the Eastern Zone of Tigray 74.65% [12]. This might be due to the difference in the study period. This may also be due to the gap of in-service training among health providers on essential newborn care. This might also be due to the difference between studies participants in which only nurses participated in the case of a study conducted in Egypt. Unavailability of materials (guidelines, drug, and vaccine) in the health facilities may hinder the knowledge of health care provider. This might also be due to the difference in a geographical location in which some of them were in the health centers and some of them are from hospitals [20].

The overall practice of health care providers was 59.8% (52.5, 65.6). This is consistent with the studies done in Bahardar 59.7%, in Vietnam 64% [20], Tigray [21]. This finding is higher than the studies done Jimma 51.1% [9] and lower than the studies done in Eastern Tigray 72.77% [12] and in Egypt 69.2% [19]. This might be due to socio-demographic difference and level of knowledge of the caregivers.

In multivariate logistic regression, unavailability of materials (guidelines, drug, etc.) in the health facilities were significant predictors of health care provider’s knowledge. Therefore, if there are limited materials (guidelines, drug, and vaccine) in the health center the health care providers cannot get enough knowledge regarding essential newborn care. Hospital management should fulfill the standard materials like guidelines, drug, and vaccine in delivery and neonatal units to all staff to increase the knowledge level of the staffs.

Those health care providers having training essential newborn care were two times more likely practicing good newborn care practice as compared to their counterpart. After performing sequential immediate newborn care steps in training place, health professions could demonstrate proficiency in the health facilities.

## Conclusion

Generally, health care providers have knowledge and practice gap. Unavailability of materials (guidelines, drug, etc.) in the health facilities and attaining of training were the factors affecting the knowledge and practice of health care providers. In order to enhance the knowledge and practice of immediate newborn care institutions should provide training and materials in the health facilities. Policymakers are also aware of this clinical gap to put possible interventions. Researchers should do other research for a strong recommendation with increasing sample sizes.

## Limitation

This study had recall bias. The study also shares the limitation of the cross-sectional study design does not show temporal cause and effects. The sample size is small and it is difficult to generalize for the population.

## Data Availability

All data is available via this manuscript.

## Abbreviations

WHO: World Health Organization
AOR: adjusted odds ratio
COR: crude odds ratio
ENC: essential newborn care
SD: standard deviation
